# Preserving Biodiversity as Source of Health Promoting Compounds: Phenolic Profile and Biological Activity of Four Varieties of *Solanum lycopersicum* L.

**DOI:** 10.3390/plants10030447

**Published:** 2021-02-26

**Authors:** Immacolata Faraone, Daniela Russo, Maria Ponticelli, Vincenzo Candido, Donato Castronuovo, Loriana Cardone, Chiara Sinisgalli, Fabiana Labanca, Luigi Milella

**Affiliations:** 1 Department of Science, University of Basilicata, via dell’Ateneo Lucano 10, 85100 Potenza, Italy; immacolata.faraone@unibas.it (I.F.); daniela.russo@unibas.it (D.R.); maria.ponticelli@unibas.it (M.P.); chiara.sinisgalli@unibas.it (C.S.); 2 Spinoff Bioactiplant, via dell’Ateneo Lucano 10, 85100 Potenza, Italy; 3 School of Agricultural, Forest, Food and Environmental Sciences, University of Basilicata, 85100 Potenza, Italy; vincenzo.candido@unibas.it (V.C.); donato.castronuovo@unibas.it (D.C.); loriana.cardone@unibas.it (L.C.)

**Keywords:** *Solanum lycopersicum* L., local cultivars, HPLC-DAD, antioxidant activity, carotenoids, phytochemical profile

## Abstract

Tomato (*Solanum lycopersicum* L.) is a precious source of specialized metabolites with a great role in human health. Many varieties of tomatoes characterize the south of Italy’s agronomic production and biodiversity, thanks to its warm temperatures and favorable weather. The preservation of biodiversity is a major goal of recent years, as each variety shows a peculiar phytochemical profile and provides a wide variety of metabolites with health-beneficial properties. Among the wide range of tomato varieties, literature focused on the most commercially-known types, including San Marzano and Datterino, while this study considered typical South Italy varieties for the first time, as well as Crovarese and Arsicolo. The aim of our work is to enrich the current knowledge about the tomato by evaluating the carotenoid content, the phytochemical profile by HPLC-DAD, and the biological activity of the different parts (peel, fruit, pulp, and seeds) of niche cultivars compared with commercial ones. Radical scavenging activity, assessed by the 2,2-Diphenyl-1-picrylhydrazyl (DPPH) method, was higher in Crovarese peel extract, while Arsicolo possessed the highest lycopene content, underlying the importance of local ecotypes as a precious source of health promoting compounds. However, out of all of the varieties considered, peel extract was the most active one, opening new insights on their valorization in light of the circular economy.

## 1. Introduction

Tomato (*Solanum lycopersicum* L.) is one of the most widespread crops worldwide, and is considered to be the sixth most valuable food crop in the world. It is largely consumed in fresh and processed forms and it is one of the staple foods in the Mediterranean Diet [1]. Tomato is a precious source of specialized metabolites, formally named as secondary metabolites, with a great role in human health. In this context, vegetables with high content of functional compounds are the key players in human health. It is estimated that around 80% of dietary lycopene is provided by tomato intake, with beneficial effects on human health [2]. Regular consumption of tomatoes and derivatives, indeed, has been associated with reduced rates of cancer and chronic diseases, thanks to its high content of carotenoids and phenolic compounds that make this fruit a significant source of natural antioxidants. Among them, the most representative carotenoids are lycopene and *β*-carotene, while rutin, quercetin, and naringin are the just a few examples of the wide range of phenolic compounds that characterize tomato fruits [1]. However, the phytochemical profile is affected by many factors, as well as variety, geographical location, and growth conditions [2]. The south of Italy provides warm temperatures which are ideal for growing tomato. Indeed, many varieties of tomatoes characterize the area, contributing to its biodiversity. The conservation of local cultivars is realized by farmers who are experts in traditional agronomic practices and in passing on this knowledge on from generation to generation [3]. Several agrobiodiversity studies have focused not only on the enrichment of nutritional profile in traditional crops [4,5,6], but also on the different susceptibility to negative plant–soil feedbacks [7], pollinators, and pest pressure. In this context, the lack of irrigation could have allowed us to obtain fruits characterized by an interesting chemical profile [8]. With their chemodiversity, these tomato varieties can contribute considerably to the local biodiversity of Campania region, in agreement with other studies who evaluated ecotypes such as ‘Vesuvio’, ‘Sorrento’, ‘San Marzano’ [5], and ‘Piennolo’ [6]. In addition, the co-existence of many varieties could be a strategy to improve the pollination services, considering that honeybees should be attracted by a specific variety rather than others [9]. Moreover, cultivated tomatoes are more vulnerable to negative plant–soil feedbacks than their wild relatives, suggesting a different impact on microbial communities, based on their variety [7]. Further, in wild tomato species, the occurrence of sucrose esters has also been associated with a better resistance to aphids. Indeed, it is hypothesized that the biodiversity is a resource for insect pest management [10]. Thus, the preservation of ancient tomato varieties would increase the vegetation diversity, positively influencing natural enemy diversity, with a positive impact on the lowering of pest pressure on tomato crops [11]. However, whilst the preservation of biodiversity is a great goal of recent years, ever-increasing market demand led to the development of new genotypes and hybrids through molecular breeding. These cultivars show different properties, as well as different phytochemical profiles and nutritional qualities, and a better adaption to the environment. Among the wide range of tomato varieties, the literature focused on the most commercially-known, including San Marzano [12] and Datterino [13], while this study considered, for the first time, typical South Italy varieties: Crovarese and Arsicolo. Local genotypes arise as important alternative sources of health-promoting compounds, as well as sources of great genetic variability, contributing significantly to local biodiversity [4,5,6]. On the other hand, the assessment of phytochemical profile and antioxidant activity of the different parts of tomatoes, mainly peel and seeds, paves the way for new progress towards the Green Economy, through the appreciation of by-products deriving from tomato working-processes. The aim of our work is to enrich the current knowledge about tomato bioactive compounds related to the cultivar by evaluating the phytochemical profile and the biological activity of the different parts (peel, seeds, pulp, and fruit) of niche cultivars (Arsicolo and Crovarese), compared with commercial ones (Datterino and San Marzano).

## 2. Results and Discussion

Tomatoes include thousands of different metabolites, in variable proportions, depending on the fruit, agricultural conditions, and variety. The major challenge is to extract the majority of them. For this reason, we performed a simple and reproducible extraction protocol using methanol as a solvent. It is able to allow a successful recovery of both polar and semipolar metabolites, with great importance for human health [14].

### 2.1. Antioxidant Activity and Phytochemical Profile

All the extracts were tested for their antioxidant activity. In vitro assays are useful to evaluate the ability of plant extracts to quench radicals. In our study, the radical scavenging activity was assessed against the synthetic 2,2-Diphenyl-1-picrylhydrazyl (DPPH) radical. All compounds in the extracts affect this activity, positively or negatively. The outcomes of the antioxidant assay on tomato samples showed a high degree of correlation with some quantified phenolics, highlighting the pivotal role of these compounds as majorly responsible for the radical-scavenging activity of tomato extracts, as described below. By contrast, carotenoids are not strongly correlated with antioxidant activity (*r* < 0.3, *p* > 0.3), confirming the assumption of George et al. [15] that carotenoids are not involved in the radical scavenging ability of extracts when it is assessed by the DPPH method. The lipophilic nature of these compounds might explain their low activity in polar systems, such as DPPH assay. The results of antioxidant activity, assessed by the DPPH method, are presented in Table 1. For DPPH assay, the values range from 11.14 ± 1.25 to 111.32 ± 11.81 mg TE/100 g FW. According to Chandra and Ramalingam [16], the highest radical scavenging activity was shown by peel extract, followed by fruits, seeds, and pulp (Table 1). Among the four varieties, Crovarese and Arsicolo (peel) showed the best antioxidant potential, with DPPH results of 111.32 ± 11.81 and 70.34 ± 7.97 mg TE/100 g FW, respectively. Lower values have been reported for the two commercial cultivars Datterino and San Marzano (peel) of 60.13 and 67.81 mg TE/100 g FW, respectively. Although recent studies of molecular analysis revealed a genetic relationship between the Crovarese and San Marzano varieties [17], their antioxidant properties and phytochemical profile are quite different, confirming the thesis of Aherne et al. [2], who demonstrated that the impact of geographical location on the phytochemical profile of tomatoes is greater than the influence of variety. Data about radical scavenging activity revealed good results for the Datterino fruit extract (86,79 ± 6.18 mg TE/100 g FW), being second only to Crovarese peel extract in terms of antioxidant activity. This could be explained by skin influence on the fruit extracts, thanks to the higher ratio between skin and fruit, due to the small size of the Datterino berry. However, in accordance with Chandra et al. [18], extract of fruit, seeds, and pulp generally did not reach the IC_50_, unlike in the case of skin extracts. This could be due to the richness in specialized metabolites positively correlated with the antioxidant activity (an *r* close to one) in peel extracts. Indeed, correlation analysis between radical scavenging activity and specialized metabolites reported the best results for compounds that mainly occur in peel extracts: narirutin (*r* = 0.78, *p* = 0.0004), 3,4-dihydroxybenzoic (*r* = 0.64, *p* = 0.008), and gallic acid (*r* = 0.52, *p* = 0.039), followed by epicatechin (*r* = 0.43, *p* = 0.093) and vanillic acid (*r* = 0.30, *p* = 0.267). Conversely, *p*-cumaric (*r* = −0.26, *p* = 0.326) and *trans*-cinnamic (*r* = 0.08, *p* = 0.779) acids exhibited the lowest DPPH scavenging values and are present only in traces in the skin extracts. Not all compounds that belong to the phenolic class possess a radical scavenging activity, as demonstrated by negative *r* values. This may be due to the nature of the radical used in the test, or structural features and steric hindrance of the different phytocomponents of the extract [19]. It should be the case for bulky molecules as rutin, the main component of Datterino fruit extract that displayed *r* = 0.22, *p* = 0.419. However, the antioxidant potential of Datterino may be attributable to other not identified compounds. A detailed description of all identified compounds is provided in Section 2.4. Although seeds are not distinguished by a particular richness of metabolites, they possess a mild antioxidant activity, arising as a valuable source of antioxidants for nutraceutical purposes. Given the high quantities of seeds and peels coming from the tomato working processes, the rich phytochemical profile, together with the antioxidant properties, remain attractive for their application as a source of health promoting compounds, shedding a light on re-use in the food industry.

### 2.2. Determination of Chlorophyll and Carotenoid Content

**Figure 1:**
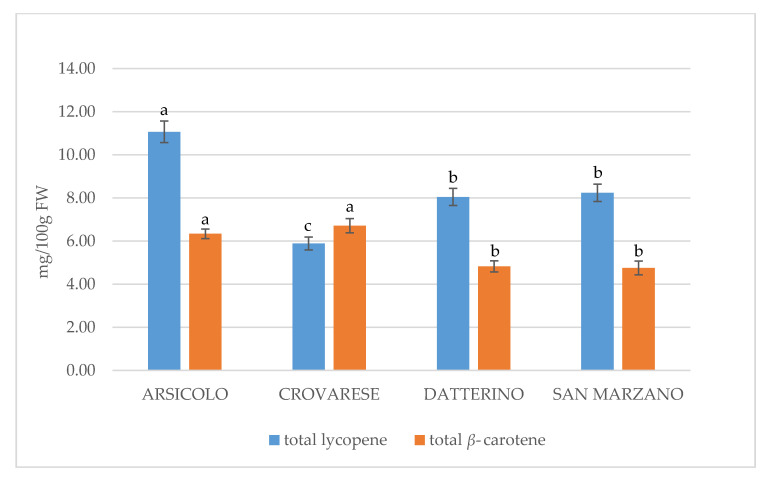
Comparation of total lycopene and *β*-carotene content among all varieties. Significant differences (*p* < 0.05), highlighted with different superscript letters (a–c), were evaluated for lycopene and *β*-carotene separately.

**Figure 2:**
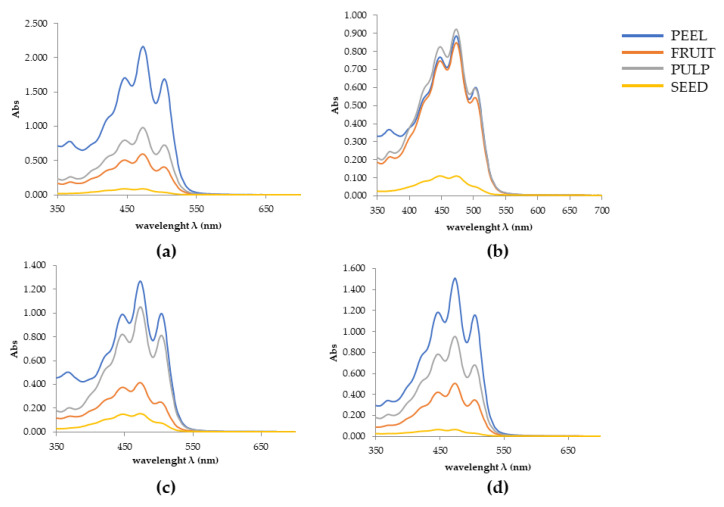
Absorbance spectra of acetone/hexane (2:3, *v/v*) extracts of different parts of tomatoes (blue, peel; orange, fruit; gray, pulp; yellow, seeds) in (**a**) Arsicolo, (**b**) Crovarese, (**c**) Datterino, and (**d**) San Marzano cultivars. The absorbance of the supernatants was recorded at 453, 505, 645, and 663 nm by SpectroStar Nano (BMG Labtech).

Chlorophylls are photosynthetic pigments with a porphyrin ring that chelated a magnesium ion and a phytol tail from the isoprenoid pathway. Two major chlorophylls (a and b) occur in all evolved photosynthetic organisms. Chlorophyll degradation is a physiological process that occurs during different phases of plant development. During tomato ripening, chlorophylls are degraded. As shown in Table 2, chlorophylls in our ripened samples are detectable in small amounts. Gene expression analysis demonstrated that the pheophorbide A oxygenase/phyllobilin pathway is mainly responsible for this process [20], with a corresponding conversion of the color from green to the typical red of tomato. The physiological breakdown of chlorophylls in favor of carotenoids is clearly shown in our study and it should be explained by the addressing of the metabolic pathway towards the increment of carotenoid synthesis, as both classes share the same precursor [21]. Many studies highlighted the chlorophyll-associated antioxidant and anticancer effects, thanks to the ability to inhibit the lipid peroxidation [22,23]; however, carotenoids are mainly responsible for the health benefits of tomato intake. Previous studies have demonstrated that carotenoid content varies between the different tomato species [2]. Also, their bioaccessibility is influenced by variety and geographical location [2]. Therefore, many studies have been oriented towards the research of new genotypes with high carotenoid content and antioxidant properties. However, intensive farming has led to the pauperization of beneficial compounds in the commercial varieties [4]. For this reason, our study investigated the content of carotenoids in both commercial (Datterino and San Marzano) and less-known varieties (Arsicolo and Crovarese). Our results are in accordance with this trend, showing that the total carotenoid content in Arsicolo peel extract was double that of Datterino, as well as the Crovarese fruit extract with respect to the same extract of San Marzano (Table 2). Our results confirmed previous findings, according to which lycopene is more abundant compared to *β*-carotene, as clearly shown in Figure 1. Moreover, compared to other carotenoids, lycopene showed the highest antioxidant activity, thanks to the conjugated double bonds that characterize its chemical structure [18]. The abundance of lycopene mostly characterizes the peel of each variety, followed by pulp, and seed, in accordance with Chandra and Ramalingam [16]. The lycopene concentration ranges from 0.1 to 6.48 mg/100 g FW. The highest value in Arsicolo skin (6.48 ± 0.52 mg/100 g FW) is statistically significant with respect to the other peel extracts. Our data fall within the ranges previously reported in the literature [1,24], although our results on the lycopene content in San Marzano variety are higher than those obtained by Berni et al. [4]. For the Datterino variety, indeed, Muratore et al. [13] estimated that the lycopene content was between 3.98 and 5.22 mg/100 g FW, in accordance with our findings. Conversely, some authors reported slightly higher concentrations in pulp of commercially available Nigerian tomatoes (12.4 ± 0.01 mg/100 mL) [25], or in organically fertilized cultivars (8–12 mg/100 g FW) [26]. This might be explained by genetic differences among varieties, as well as the physiological maturity. Tomato fruits belonging to the same cultivar indeed could show significant differences in lycopene concentration, depending on grade of ripeness [27]. Thus, genotype and environmental conditions, including temperatures and altitude, affect the concentration of lycopene. As demonstrated by Chandra et al. [18], cultivars growing at higher altitudes increased the lycopene content, while cultivars exposed to very hot temperatures and strong solar irradiation decreased it. In these conditions, tomatoes tend to develop a more yellow color, as lycopene is accountable for the redness of tomato. Following the same trend, *β*-carotene is also more detectable in skin than in pulp and seeds. However, differently from lycopene, the differences among the four parts are less pronounced, as shown in Figure 2. The highest value was found for Arsicolo peel, confirming this local cultivar as being the richest in carotenoids. Comparing with previous literature results [28,29], our data for *β*-carotene content are generally higher. We found only slight similarities with an ancient variety analyzed by Kaur et al. [28], demonstrating, once again, the intrinsic value of ancient cultivars in terms of nutraceuticals, with respect to the commercial varieties. Although it constitutes a small part of total carotenoids, the nutritional importance of *β*-carotene is widely recognized. Thanks to the provitamin A activity, it exerts a positive impact in many biological processes, involving vision, bone development, and skin health [28], as well as oxidative stress defense.

### 2.3. HPLC-DAD Analysis

Tomatoes are characterized by a high nutritional value, thanks to their wide content of natural antioxidants like carotenoids, vitamin C, and phenolic compounds. The growing interest in tomato antioxidant power and related health benefits might explain research attempts to identify the main phytocomponents that should be responsible for the tomato’s properties.

**Figure 3:**
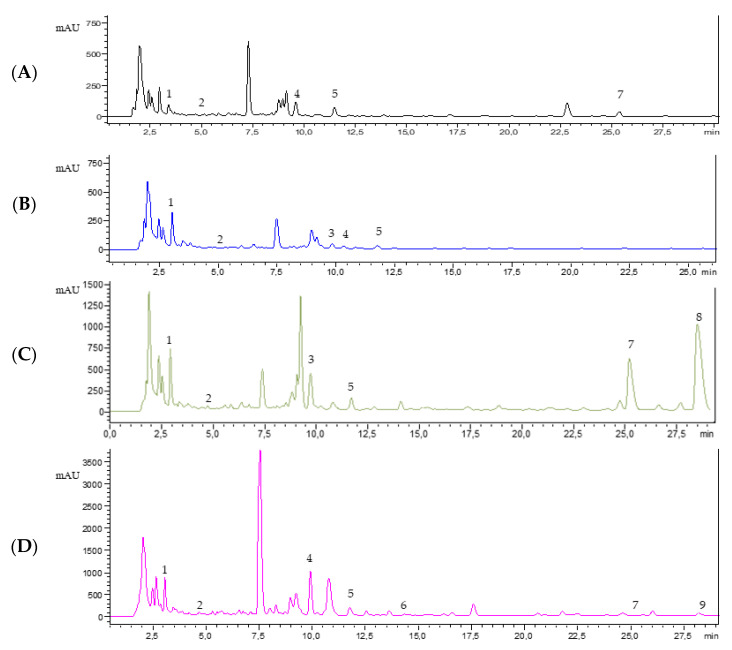
Exemplificative HPLC-DAD chromatogram of extracts from four parts fruit (black); pulp (blue); peel (green); seeds (violet) of San Marzano tomato (280 nm). Identified compounds are the following based on Retention Time (RT): (**A**) 1, gallic acid = 3.42 min; 2, 3,4-dihydroxybenzoic acid = 4.77 min; 4, caffeic acid = 9.60 min; 5, epicatechin = 11.48 min; 7, narirutin = 22.39 min; (**B**) 1, gallic acid = 3.04 min; 2, 3,4-dihydroxybenzoic acid = 4.82 min; 3, vanillic acid = 9.19 min; 4, caffeic acid = 10.30; 5, epicatechin = 11.77 min; (**C**) 1, gallic acid = 3.03 min; 2, 3,4-dihydroxybenzoic acid = 4.82 min; 3, vanillic acid = 9.19 min; 5, epicatechin = 11.83 min; 7, narirutin = 25.04 min; 8, naringin = 28.65 min; (**D**) 1, gallic acid = 3.07 min; 2, 3,4-dihydroxybenzoic acid = 4.69 min; 4, caffeic acid = 9.92 min; 5, epicatechin = 11.77 min; 6, *p*-coumaric acid = 14.34 min; 7, narirutin = 25.26 min; 9, rutin = 28.40 min.

**Figure 4:**
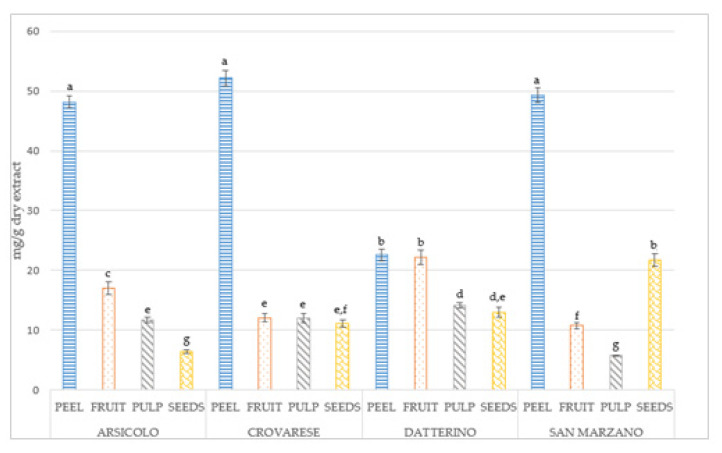
Distribution of quantified specialized metabolite content (mg/g dry extract) among the four different parts. Significant differences (*p* < 0.05), calculated between all samples of the same line, are highlighted with different superscript letters (a–g).

The DAD allows us to record the UV/Vis spectra of every compound, attributing each peak of the chromatogram to a specific class of metabolites based on the absorbance maximum and characteristic spectrum that every molecule exhibits thanks to chromophores. Results of the phytochemical profile shown by HPLC-DAD for each tomato sample are summarized in Table 3, while Figure 3 shows an exemplificative chromatogram obtained from analysis of the extracts of four parts of San Marzano. In accordance with antioxidant activity results, HPLC quantification revealed the highest content of specialized metabolites (mg/g dry extract) in peel extracts, although not statistically significant quantitative differences could be detected among the four varieties (Table 3, Figure 4). We identified and quantified a total of 11 compounds, 6 phenolic acids, and 5 flavonoids. The main phenolic compounds identified in our study are almost the same as the ones found in previous studies [19,30], and in comparable levels. A separate discussion is deserved for the results of Szabo et al. [30], who considered 10 tomato varieties processing waste, mainly constituted by peels, with much lower values than those obtained in our peel extracts. This might have been due to thermal processing causing the degradation of some compounds. However, slight differences occur in terms of most abundant compounds: our results reported that gallic acid was the most concentrated phenolic acid, followed by vanillic acid, while Valdez-Morales et al. [19] found the highest concentrations in caffeic acid and Barros et al. [31] in *cis p*-coumaric acid. These hydroxycinnamic acids are present only in traces in our extract, reflecting a characteristic composition for each tomato cultivar, related to genetic nature and environmental conditions. Regarding flavonoids, our results agree with the previous literature [19,31]. The most abundant flavonoid was narirutin, with 27.65 mg/g dry extract for Crovarese peel extract. It is also the richest extract in gallic acid (18.32 mg/g dry extract), while Arsicolo peel extract presented the highest detected value of vanillic acid (13.33 mg/g). This peculiar phytochemical profile might explain the better results shown in DPPH assay. Differently, other literature sources reported that naringenin derivatives [32] and kaempferol glucosides [33] are the main components of tomato extracts. Regarding other chemical classes, alkaloids and terpenes are often present in unprocessed tomatoes [34]. However, the focus of our research was the quali-quantitative analysis of bioactive compounds reported in tomato parts, that are often discarded and are mostly responsible for the antioxidant activity. Consequently, we focused our attention on phenolic metabolites, which are most abundant in peel and seeds [34,35]. The large variety among the different species of tomatoes reflects the influence of different genotype in the phytochemical composition. Indeed, the chemical profile might be considered a variety and cultivar distinctive factor in plants, depending on the genetic and environmental conditions [31]. As we considered niche cultivars that are locally distributed, it was not possible to find information about chemical composition of Arsicolo and Crovarese varieties, thereby enriching the present work with a great novelty. Regarding San Marzano, the phytochemical profile was evaluated by Berni, et al. [4] with lower levels of phenolic compounds compared to those reported in our study for the same variety. Due to the great potential of traditional crops that emerged from our study, the use of untargeted metabolic analysis should be useful in follow-up studies in order to have a full scan of all tomato components and to complete the picture, as these traditional varieties are under-researched.

### 2.4. Principal Component Analysis (PCA)

**Figure 5:**
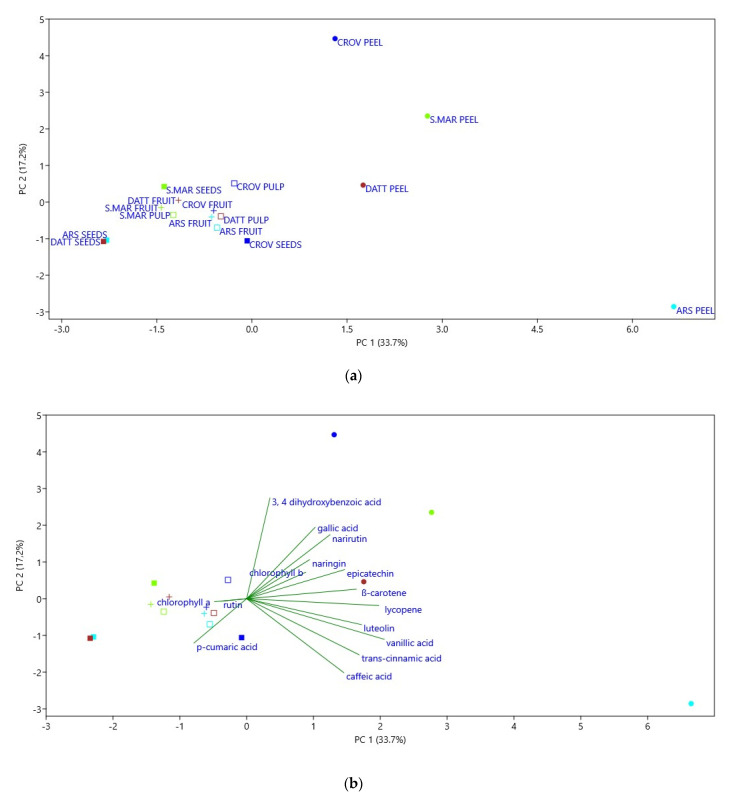
Results of PCA analysis. Score plot (**a**) and the loading plot (**b**) of the variables are reported. The two principal components account for 50.9% of the total variance, 33.7% of which involves PC1 and 17.2% involves PC2. PCA showed a clear discrimination among peel extracts and other tomato extracts.

The use of PCA to assess the metabolomic differences between plant varieties has been widely validated [36]. The results regarding the different tomato parts are shown in Figure 5, where the score plot and the loading plot of the variables are reported. The two principal components account for 50.9% of the total variance, 33.7% of which involves PC1 and 17.2% involves PC2. In accordance with antioxidant results, PCA showed a clear discrimination among peel extracts and other tomato extracts (pulp, fruit, and seeds). In particular, peel extracts are positively correlated to all horizontal factors that are addressed towards them, but Arsicolo peel seems to be clustered separately, correlating with variables directed towards it. By contrast, the other tomato portions, independently from variety, are closely grouped, underlying small differences among them, mostly in terms of specialized metabolite content. The loading plot also highlights that the strongest antioxidant activity of peel extracts is directly correlated to the abundance of specific bioactive compounds, as demonstrated by the proximity of peel spots to corresponding compound vectors. Thus, the PCA results indicated the strength of the study, demonstrating that differences among phytochemical composition depend on genetic differences and environmental conditions.

## 3. Materials and Methods

### 3.1. Chemicals

Analytical grade methanol was obtained from Merck (Darmstadt, Germany and Mollet del Vallés, Spain); HPLC grade water (18 mΩ) was prepared by a Mill-Ω purification system (Millipore Corp., Bedford, MA, USA). 2,2-diphenyl-1-picryl hydrazyl (DPPH) in free radical form, Trolox (6-hydroxy-2,5,7,8-tetramethylchroman-2-carboxylic), and the standards of phenolic compounds were purchased from Sigma (St. Louis, MO, USA and Steinheim, Germany).

### 3.2. Cultivars

The field experiment was carried out in Buccino (Salerno) (40°38′ N and 15°23′ E, 663 m a.s.l.), located in the Campania region (Southern Italy) during spring–summer of 2016. The soil was ploughed to a depth of 25 cm, fertilized by incorporating legume green manure, and then rotavated and leveled. Prior to the experiment, seeds of commercial tomato cultivars (Datterino and San Marzano) were purchased from Semiorto Sementi, while traditional cultivar seeds (Crovarese and Arsicolo) were harvested and stored by a local farmer in the same year. Seeds of all studied tomato varieties were sowed into 91-cell plastic trays filled with 80% of peat and 20% of vermiculite (*v/v*) at the end of April. The trays were transferred to a climatic chamber (25 °C for 36 h) and then to a greenhouse until the transplant. The transplantation took place at the end of May in a randomized complete block design with three replications using plants at the stage of 3rd–4th true leaf. The plants were set at a distance of 40 cm in single rows spaced 90 cm, thus obtaining a plant density of 2.78 plants/m^2^. Plants were grown under the same standard tomato field procedures of this area: for the whole tomato breeding, no irrigation was applied and organic weed control was taken. Freshly harvested and uniformly ripened healthy fruits, at the red ripe stage were used for the extraction process.

### 3.3. Extracts

Twenty-one tomatoes from each variety were harvested at full ripeness, refrigerated and immediately processed. From three of them, peel, pulp, and seeds were separated. Then, three whole tomatoes and previously obtained each separated part were extracted, this procedure was repeated three times. The extraction process was carried out in the dark to avoid the potential degradation of specialized metabolites. Samples were ground using a mortar and pestle by using liquid nitrogen, then homogenized and macerated in methanol (sample:solvent ratio 1:10) in amber bottles, covered by an aluminum foil, under agitation, in the dark, at room temperature for 1 h and then filtered [14]. The obtained extracts have been used for radical scavenging activity and HPLC-DAD analysis. For carotenoid determination, a different procedure was carried on, by extracting tomato samples (1 g from three different tomatoes per cultivar) with 15 mL of acetone/hexane (2:3, *v*/*v*).

### 3.4. Antioxidant Activity: 2,2-Diphenyl-1-picrylhydrazyl (DPPH)

Radical-scavenging activity was evaluated by 2,2-Diphenyl-1-picrylhydrazyl (DPPH) assay, as reported by Russo et al. [37]. Various dilutions of each tomato extract (50 µL) were added to 250 µL of DPPH methanol solution (100 mM) in a 96 well plate. The mixture was shaken vigorously and left at the dark after 30 min. The absorbance was read at 515 nm by using a spectrophotometer and the results were expressed as mg Trolox Equivalent/100 g of fresh weight (mg TE/100 g FW).

### 3.5. Determination of Chlorophyll and Carotenoid Content

The determination of *β*-carotene and lycopene was determined according to the method reported by Vinha et al. [35]. Following the extractive procedure mentioned above, the absorbance of the supernatants at 453, 505, 645, and 663 nm were registered by SpectroStar Nano (BMG Labtech). The content of *β*-carotene and lycopene was calculated as reported by following equations and then expressed in mg per 100 g of sample:

*β*-carotene (mg/100 mL) = 0.216 × *A*_663_ − 1.220 × *A*_645_ − 0.304 × *A*_505_+ 0.452 × *A*_453_ (1)

lycopene (mg/100 mL) = −0.0458 × *A*_663_ + 0.204 × *A*_645_ + 0.372 × *A*_505_ − 0.806 × *A*_453_. (2)

During the extraction process, the room luminosity was reduced and glass materials were covered by aluminum foil to avoid the carotenoid loss by photo-oxidation.

This extraction method was used for simultaneous determination of chlorophyll-a and chlorophyll-b by using the following equations:

Chlorophyll-a (mg/100 mL) = 0.999 × *A*_663_ − 0.0989 × *A*_645_ (3)

Chlorophyll-b (mg/100 mL) = −0.328 × *A*_663_ + 1.77 × *A*_645_. (4)

### 3.6. HPLC-DAD Analysis

The HPLC-DAD analysis of specialized metabolites was performed using a reversed-phase system (Shimadzu LC-20AB, Prominence Diode Array Detector, Shimadzu Corporation, Japan), equipped with a binary pump. The column was a Macherey-Nagel C18 column (4.6 mm × 250 mm × 5 μm). The wavelength was set at 254 nm, and the analysis time was 70 min. Two different solvents were used as a mobile phase: A (0.05% formic acid aqueous solution) and B (methanol) programmed in a gradient as follows: 0 min (5% B); 5 min (15% B); 18 min (25% B); 35 min (35% B); 50–60 min (100% B); 65–70 min (5% B). Each mobile phase was filtered through a 0.20 μm membrane filter before use. The samples were dried by a rotavapor an solubilized in water at a concentration of 5 or 10 mg/100 μL. Samples (20 μL) were injected at a flow rate of 0.60 mL min^−1^. The system was allowed to equilibrate for approximately 10 min for the subsequent injection. The identification of each compound was obtained according the retention time and the UV-Visible spectra of the corresponding standard compounds. The linearity of the standard was calculated by taking the peak area as a function of the concentration and expressed as mg of standard/kg of dry extract and was converted into mg of standard/100 g FW. The experiments were repeated three times and the results expressed as mean ± standard deviation.

### 3.7. Statistical Analysis

All experiments were repeated in triplicate and results expressed as mean ± standard deviation. By using GraphPad Prism 5 (La Jolla, CA, USA), the analysis of variance (one way ANOVA) was performed to assess statistically significant differences among samples at a confidence level of 95%. Differences on the mean values were assessed by the Tukey test at a significance level of *p* < 0.05. Moreover, in order to test the ANOVA requirement for data normal distribution, the Shapiro Wilk test was used (*p* < 0.05) and to assess the relationships among the variables, the correlation analysis (Pearson’s *r*) was performed. This latter is calculated by dividing the covariance (*cov*) of the two variables (*x, y*) by the product of their standard deviations (*s*), as in the following equation:

$$r=\;r_{x,y}\;\frac{cov\;\left(x,y\right)}{s_{x}\;s_{y}}\;.$$

The value of *r* ranges between −1 and 1. A correlation of −1 shows a perfect negative correlation, while a correlation of 1 shows a perfect positive correlation. A correlation of 0 shows no relationship between the two variables.

Principal component analysis (PCA), a multivariate statistical tool was also performed on the data by using the statistical package Statistica for Windows (ver. 5.1., 1997) (Statsoft Inc., Tulsa, OK, USA) [38]. PCA, through a *n*-dimensional vector approach, divides samples basing on the cumulative correlation of all component data and then identifies the compounds exhibiting the greatest variance within a population, determining those with a greater correlation. The mean values have been used for PCA by applying a correlation analysis.

## 4. Conclusions

The renewed interest towards local cultivars derived from the will to preserve biodiversity and local identity. Besides this, the geographical conditions, together with the genomic traits, influence the organoleptic features of local varieties, thanks to the different phytochemical profile. Although many cultivars have been set aside over the years, due to having the lowest yield or the complexity of growth conditions, recently they have been rediscovered for the peculiarity of their composition. Precious bioactive molecules are differently distributed among the wide variety of cultivars, affecting the nutritional value of each specie. Indeed, in our work, differences between local and commercial varieties have been highlighted in terms of specialized metabolite profile, mainly phenolic and carotenoids, and their correlation with the antioxidant activity has been investigated. From our results, it emerged that extracts from local varieties (Arsicolo and Crovarese) possessed the best antioxidant and radical scavenging activity, thanks to the abundance of bioactive compounds with well-known antioxidant potential. Most of them belong to the phenolic class, as well as gallic acid, vanillic acid, 3,4-dihydroxybenzoic acid, caffeic acid, and narirutin that are among the principal compounds identified in our study. Arsicolo and Crovarese extracts also revealed to be the richest in *β*-carotene, while the Arsicolo content of lycopene is the highest among all extracts. The highest antioxidant capacity in Crovarese and Arsicolo varieties might be due to the fact that local cultivars have been less genetically manipulated than commercial ones [19], where genetic improvement to increase the production has led to a loss of some metabolites and, then, loss of bioactivity, as demonstrated by Prohens, et al. [39] in eggplants and ancestors, belonging to the same genus. The cultivation of tomato has also had a negative impact on the fruit organoleptic properties. As demonstrated by Cortina, et al. [40], when we compare commercial cultivars with traditional ones, we can found a lot of differences in terms of chemical composition, mainly in volatile organic compounds that are responsible for typical flavors and odors. All of these differences contribute to the chemodiversity of tomato, further confirmed by our study that highlighted the different occurrence of specialized metabolites among commercial and local cultivars. Nevertheless, further studies will be performed to have a complete overview of the quali-quantitative phytochemical profile differences by investigating the volatile fraction, rather than the volatile terpenoid and alkaloid contents. These local varieties might have the potential to satisfy the ever-increasing requirement for food products with high nutraceutical compound content. In a long-term vision, the valorization of these niche cultivars could encourage their commercialization, with economic advantages for the small local areas. Peel extracts were demonstrated to be more active with respect to other tomato parts. This evidence shed a light on their employment as economic source of health-promoting compounds, given the high quantities of peels coming from the tomato sauce working processes. The rich phytochemical profile, together with the antioxidant properties, remain attractive for their industrial application in nutraceutical and medicinal fields.

## Figures and Tables

**Table 1:** Results of radical scavenging activity, assessed by 2,2-Diphenyl-1-picrylhydrazyl (DPPH) method for four parts (peel, fruit, pulp, and seeds of four different tomato cultivars (Arsicolo, Crovarese, San Marzano, and Datterino)).

Samples	DPPH (mg TE/100 g FW)
ARSICOLO PEEL	70.34 ± 7.97 ^c^
ARSICOLO FRUIT	30.44 ± 3.18 ^f^
ARSICOLO PULP	11.14 ± 1.25 ^i^
ARSICOLO SEEDS	18.33 ± 1.7 ^h^
CROVARESE PEEL	111.32 ± 11.81 ^a^
CROVARESE FRUIT	40.38 ± 4.66 ^e^
CROVARESE PULP	25.98 ± 3.44 ^g^
CROVARESE SEEDS	38.32 ± 2.18 ^e^
DATTERINO PEEL	60.13 ± 3.88 ^c^
DATTERINO FRUIT	86.79 ± 6.17 ^b^
DATTERINO PULP	41.7 ± 4.27 ^e^
DATTERINO SEEDS	50.4 ± 5.93 ^d^
SAN MARZANO PEEL	67.81 ± 5.94 ^c^
SAN MARZANO FRUIT	54.88 ± 3.08 ^d^
SAN MARZANO PULP	39.03 ± 1.73 ^e^
SAN MARZANO SEEDS	42.03 ± 3.28 ^e^

Results are expressed as mean ± standard deviation of mg of Trolox Equivalents (TE)/100 g of fresh weight (FW). Significant differences (*p* < 0.05) between all samples are highlighted with different superscript letters (a–i).

**Table 2:** Carotenoid and chlorophyll content of four parts (peel, fruit, pulp, and seeds) of four different tomato cultivars (Arsicolo, Crovarese, San Marzano, and Datterino).

Samples	mg *β*-Carotene/100 g FW	mg Lycopene/100 g FW	mg Chlorophyll a/100 g FW	mg Chlorophyll b/100 g FW
ARSICOLO PEEL	2.98 ± 0.15 ^a^	6.48 ± 0.52 ^a^	0.04 ± 0.00 ^c^	0.06 ± 0.008 ^d,e^
ARSICOLO FRUIT	1.18 ± 0.09 ^e,f^	1.39 ± 0.10 ^f^	0.01 ± 0.00 ^f^	0.04 ± 0.005 ^f,g^
ARSICOLO PULP	1.83 ± 0.13 ^b–d^	3.10 ± 0.26 ^c^	0.03 ± 0.00 ^d^	0.04 ± 0.005 ^f,g^
ARSICOLO SEEDS	0.35 ± 0.05 ^h^	0.10 ± 0.01 ^h^	0.04 ± 0.01 ^c^	0.06 ± 0.007 ^d,e^
CROVARESE PEEL	1.89 ± 0.18 ^b,c^	2.01 ± 0.20 ^d,e^	0.03 ± 0.01 ^d^	0.05 ± 0.01 ^e,f^
CROVARESE FRUIT	2.06 ± 0.22 ^b,c^	1.75 ± 0.15 ^e,f^	0.04 ± 0.00 ^c^	0.03 ± 0.00 ^g,h^
CROVARESE PULP	2.34 ± 0.20 ^b^	1.99 ± 0.26 ^d,e^	0.06 ± 0.01 ^a^	0.09 ± 0.01 ^b,c^
CROVARESE SEEDS	0.43 ± 0.05 ^h^	0.14 ± 0.02 ^h^	0.05 ± 0.01 ^b^	0.06 ± 0.01 ^d,e^
DATTERINO PEEL	1.56 ± 0.13 ^c–e^	3.76 ± 0.45 ^b^	0.03 ± 0.01 ^d^	0.06 ± 0.01 ^d,e^
DATTERINO FRUIT	1.29 ± 0.10 ^d–f^	0.92 ± 0.10 ^g^	0.01 ± 0.00 ^f^	0.01 ± 0.01 ^i^
DATTERINO PULP	1.35 ± 0.10 ^d–f^	3.13 ± 0.29 ^c^	0.02 ± 0.00 ^e^	0.06 ± 0.01 ^d,e^
DATTERINO SEEDS	0.63 ± 0.05 ^g,h^	0.24 ± 0.03 ^h^	0.01 ± 0.00 ^f^	0.02 ± 0.00 ^h^
SAN MARZANO PEEL	1.91 ± 0.20 ^b,c^	4.28 ± 0.50 ^b^	0.05 ± 0.00 ^b^	0.07 ± 0.01 ^c,d^
SAN MARZANO FRUIT	1.01 ± 0.12 ^f,g^	1.43 ± 0.14 ^f^	0.06 ± 0.01 ^a^	0.13 ± 0.02 ^a^
SAN MARZANO PULP	1.64 ± 0.17 ^c–e^	2.42 ± 0.38 ^d^	0.01 ± 0.00 ^f^	0.02 ± 0.00 ^h^
SAN MARZANO SEEDS	0.2 ± 0.02 ^h^	0.11 ± 0.01 ^h^	0.06 ± 0.01 ^a^	0.10 ± 0.02 ^b^

Results are expressed as mean ± standard deviation of mg/100 mL of fresh weight (FW). In each column, significant differences (*p* < 0.05), between all samples of the same column are highlighted with different superscript letters (a–i).

**Table 3:** Quantification of compounds (indicated as numbers in the first column) in *Solanum lycopersicum* L.

	SAMPLES															
	AS	AF	AP	ASD	CS	CF	CP	CSD	DS	DF	DP	DSD	SMS	SMF	SMP	SMSD
**1**	8.57 ± 0.69 ^c,d^	7.89 ± 0.45 ^c,d^	9.65 ± 0.70 ^b,c^	3.34 ± 0.23 ^g,h^	18.32 ± 1.48 ^a^	8.25 ± 0.52 ^c,d^	7.48 ± 0.33 ^d,e^	3.14 ± 0.12 ^h^	11.93 ± 0.94 ^b^	4.67 ± 0.24 ^f^	4.48 ± 0.25 ^f^	4.55 ± 0.38 ^f^	7.2 ± 0.55 ^d,e^	6.3 ± 0.43 ^e^	3.94 ± 0.25 ^f,g^	3.74 ± 0.45 ^g,h^
**2**	13.33 ± 0.80 ^a^	0.89 ± 0.05 ^h^	nd	0.64 ± 0.03 ^i^	1.02 ± 0.1 ^g,h^	1.06 ± 0.09 ^f,g^	1.42 ± 0.08 ^e^	2.54 ± 0.09 ^c^	1.91 ± 0.08 ^d^	1.28 ± 0.05 ^e^	1.24 ± 0.05 ^e,f^	0.261 ± 0.01 ^j^	4.23 ± 0.20 ^b^	nd	0.56 ± 0.03 ^i^	nd
**3**	nd	nd	nd	nd	2.38 ± 0.14 ^a^	0.04 ± 0.00 ^f^	0.71 ± 0.03 ^b,c^	0.41 ± 0.02 ^c,d^	0.93 ± 0.08 ^b^	0.39 ± 0.02 ^c,d^	0.39 ± 0.02 ^c,d^	0.28 ± 0.00 ^d^	0.77 ± 0.06 ^b^	0.13 ± 0.00 ^e^	0.42 ± 0.03 ^c,d^	1.08 ± 0.87 ^b^
**4**	1.08 ± 0.09 ^a^	0.07 ± 0.01 ^f^	0.31 ± 0.00 ^b^	nd	nd	0.05 ± 0.00 ^g^	0.14 ± 0.02 ^d^	0.28 ± 0.01 ^b^	nd	0.07 ± 0.01 ^f^	0.30 ± 0.02 ^b^	0.15 ± 0.03 ^d^	nd	0.20 ± 0.01 ^c^	0.12 ± 0.04 ^d^	0.1 ± 0.00 ^e^
**5**	3.39 ± 0.24 ^c^	2.32 ± 0.01 ^e,f^	1.35 ± 0.09 ^g^	1.05 ± 0.08 ^h^	2.65 ± 0.42 ^d,e^	2.06 ± 0.35 ^f^	2.08 ± 0.13 ^f^	3.59 ± 0.02 ^c^	4.37 ± 0.25 ^b^	3.5 ± 0.09 ^c^	2.82 ± 0.04 ^d^	1.05 ± 0.1 ^h^	5.59 ± 0.35 ^a^	0.20 ± 0.04 ^k^	0.74 ± 0.1 ^i^	0.44 ± 0.03 ^j^
**6**	17.97 ± 1.24 ^a,b^	3.99 ± 2.44 ^c,d^	nd	0.48 ± 0.17 ^e^	27.65 ± 5.77 ^a^	0.45 ± 0.15 ^e^	nd	nd	1.52 ± 1.14 ^d^	7.43 ± 3.55 ^c^	nd	5.74 ± 1.44 ^c^	17.64 ± 1.65 ^a,b^	3.9 ± 0.64 ^c,d^	nd	14 ± 1.54 ^b^
**7**	1.4 ± 0.67 ^b^	1.2 ± 0.36 ^b^	nd	nd	nd	nd	nd	nd	nd	nd	nd	nd	13.42 ± 5.44 ^a^	nd	nd	nd
**8**	nd	nd	nd	nd	nd	nd	nd	nd	0.45 ± 0.03 ^c^	4.69 ± 0.05 ^a^	4.73 ± 0.03 ^a^	nd	nd	nd	nd	1.39 ± 0.1 ^b^
**9**	1.1 ± 0.10 ^a^	0.20 ± 0.02 ^f^	nd	nd	0.13 ± 0.08 ^f^	0.12 ± 0.01 ^f^	0.10 ± 0.00 ^f^	0.54 ± 0.03 ^c,d^	0.63 ± 0.05 ^b,c^	nd	nd	0.02 ± 0.00 ^f^	0.43 ± 0.03 ^d^	nd	nd	0.74 ± 0.07 ^b^
**10**	0.24 ± 0.01 ^b,c^	0.25 ± 0.01 ^b^	0.25 ± 0.01 ^b^	0.69 ± 0.44 ^a^	nd	0.05 ± 0.00 ^d^	0.07 ± 0.00 ^d^	nd	0.25 ± 0.01 ^b^	0.17 ± 0.01 ^b,c^	0.16 ± 0.00 ^c^	0.89 ± 0.07 ^a^	nd	nd	nd	0.26 ± 0.01 ^b^
**11**	1.03 ± 0.05 ^a^	0.20 ± 0.01 ^c^	0.14 ± 0.00 ^d^	0.14 ± 0.01 ^d^	nd	nd	nd	0.55 ± 0.02 ^b^	0.61 ± 0.05 ^b^	nd	nd	nd	nd	nd	nd	nd
**TOT**	48.17 ± 3.89 ^a^	17.02 ± 3.36 ^c,d^	11.71 ± 0.80 ^e,f^	6.33 ± 0.96 ^g^	52.15 ± 7.99 ^a^	12.08 ± 1.12 ^e,f^	12.01 ± 0.59 ^e,f^	11.05 ± 0.31 ^f^	22.59 ± 2.63 ^b^	22.20 ± 4.02 ^b,c^	14.12 ± 0.41 ^d,e^	12.95 ± 1.94 ^d–f^	49.28 ± 8.28 ^a^	10.73 ± 1.12 ^f^	5.78 ± 0.45 ^g^	21.71 ± 3.07 ^b,c^

Numbers correspond to compound quantification as follows; results are expressed as mean ± standard deviation of mg/g dry extract of (1) gallic acid; (2) vanillic acid; (3) 3,4-dihydroxybenzoic acid; (4) caffeic acid; (5) epicatechin; (6) narirutin; (7) naringin; (8) rutin; (9) luteolin; (10) *p*-cumaric acid; (11) *trans*-cinnamic acid in Arsicolo skin (AS), pulp (AP), fruit (AF), and seeds (ASD); Crovarese skin (CS), pulp (CP), fruit (CF), and seeds (CSD); Datterino skin (DS), pulp (DP), fruit (DF), and seeds (DSD); San Marzano skin (SMS), pulp (SMP), fruit (SMF), and seeds (SMSD). The quantification of compounds was calculated as the sum of the peak area detected at the optimal wavelength (280, 320, 350, 365 nm). Significant differences (*p* < 0.05), calculated between all samples of the same line, are highlighted with different superscript letters (a–k).

## Data Availability

Data will be available upon request.
